# The Coronavirus Pandemic (SARS-CoV-2): New Problems Demand New Solutions, the Alternative of Mesenchymal (Stem) Stromal Cells

**DOI:** 10.3389/fcell.2020.00645

**Published:** 2020-07-16

**Authors:** Noemi Eiro, Jorge Ruben Cabrera, Maria Fraile, Luis Costa, Francisco J. Vizoso

**Affiliations:** ^1^Research Unit, Fundación Hospital de Jove, Gijón, Spain; ^2^Foundation for Research With Uterine Stem Cells - FICEMU, Gijón, Spain

**Keywords:** COVID-19, SARS-CoV-2, mesenchymal stromal cell, extracellular vesicles, exosomes

## Abstract

Mesenchymal (stem) stromal cells (MSC) can be a therapeutic alternative for COVID-19 considering their anti-inflammatory, regenerative, angiogenic, and even antimicrobial capacity. Preliminary data point to therapeutic interest of MSC for patients with COVID-19, and their effect seems based on the MSC’s ability to curb the cytokine storm caused by COVID-19. In fact, promising clinical studies using MSC to treat COVID-19, are currently underway. For this reason, now is the time to firmly consider new approaches to MSC research that addresses key issues, like selecting the most optimal type of MSC for each indication, assuming the heterogeneity of the donor-dependent MSC and the biological niche where MSC are located.

## Introduction

The emergence of the coronavirus pandemic (SARS-CoV-2), due to its unusual and unforeseen virulence, is demanding new strategies from all areas of society around the world. However, medical science takes the center of the stage in this crisis. Truth is we are witnessing the failure of treatments in some COVID-19 patients together with the collapse of the healthcare activity as a result of the pandemic, which is causing an unprecedented and devastating situation in the economic and social spheres.

Faced with this new global emergency situation, the world of science and medicine must react and take the example of the extraordinary effort and dedication of our healthcare personnel. The answer to a medical problem cannot be limited to the necessary but conventional mass production of respirators and ventilators, or to obtaining a vaccine, that experts assure it will not be available any time soon. Therefore, the current situation forces us to search bravely among new paradigms of science for new therapeutic alternatives.

COVID-19 mainly affects lungs. Lung tissue represents a sociology of various cell types comprising alveoli, blood and lymphatic vessels, nerves, and connective tissue. Lung is an organ directly exposed to the environment, facing microorganisms, toxic agents and pollutants. Therefore, the necessary gas exchange for organic life requires sacrifice, wear, tear and constant renewal. Proof of this is the enormous incidence of lung diseases that secularly affected humanity. Chronic obstructive pulmonary disease alone is estimated to affect more than 500 million people worldwide today.

In basal conditions there is a varied infiltrate of inflammatory cells protecting lungs, such as alveolar macrophages. However, their protection is not always sufficient against all the aggressions suffered by lungs through life, especially in children and the elderly. On the contrary, sometimes these defense mechanisms overreact in the presence of harmful agents, causing an inflammatory hyper-response that becomes more harmful that the initial aggression, due to its devastating action on tissues. In this sense, the over-activated pulmonary macrophages and the consequent “storm” of inflammatory cytokines have been identified as one of the key elements in the fatal outcome of coronavirus infection. This over-reactive response against a viral infection is a catastrophic situation and available therapeutic agents are not demonstrating the necessary efficacy. Therefore, new therapeutic strategies are urgently need. For these reasons, researchers from different branches of biomedicine have been attracted to find therapeutic alternatives for the management of this pandemic. In this context, Mesenchymal (stem) stromal cells (MSC) have been identified as an alternative capable of controlling the cytokine storm and the over-reacting immune response (Bari et al., 2020; Chen X. et al., 2020; Golchin et al., 2020; Khoury et al., 2020; Lightner and García-Olmo, 2020; Metcalfe, 2020; Zhao, 2020).

## COVID-19

Coronavirus disease 2019 (COVID-19) is a severe acute respiratory illness caused by the agent Severe Acute Respiratory Syndrome Coronavirus 2 (SARS-CoV-2) (Munster et al., 2020; Sohrabi et al., 2020). SARS-CoV-2 is a coronavirus (CoV) similar to the Severe Acute Respiratory Syndrome Coronavirus (SARS-CoV) and the Middle East Respiratory Syndrome Coronavirus (MERS-CoV).

By the end of 2019, the COVID-19 pandemic originated in Wuhan, China, and rapidly spreaded worldwide. On March 11, 2020, the World Health Organization (WHO) defined COVID-19 as a pandemic (WHO, 2020). Although of uncertain origin, the SARS-CoV-2 is transmitted from humans to humans through respiratory droplets or contaminated surfaces (Chen Y. et al., 2020; Zhu et al., 2020). Its maximum incubation period has been assumed to be from 2 weeks (January 2020) (Backer et al., 2020) to 8 weeks (Baud et al., 2020).

Clinical manifestations of the COVID-19 varies from asymptomatic or mild-disease with fever, cough or shortness of breath (81% of cases), to respiratory failure that requiring mechanical ventilation (14% of cases) and to multiple organ dysfunction syndromes (5% of cases) (Wu and McGoogan, 2020). The mortality rate ranges from 0.7% (Ji et al., 2020) to 1.5⋅2% (Baud et al., 2020).

Although COVID-19 pathogenesis is not correctly characterized yet, we already know some relevant aspects related to the damage caused by the viral infection and the immune response triggered by this virus. For cell entry, SARS-CoV-2 uses the Angiotensin-Converting Enzyme II (ACE2) receptor and the serine protease TMPRSS2 for S protein priming (Zhou P. et al., 2020), both located on the alveolar type II cells and capillary endothelium of the lungs. This fact explains, in part, that COVID-19 especially affects lungs, which are the first organ exposed to the virus due to its mode of transmission, and which have a very slow turnover for regeneration. In addition, ACE2 is expressed in other organs such as heart and kidney. This may shed some light on why some patients with COVID-19 suffer multiple organ dysfunction syndromes (Hamming et al., 2004).

**FIGURE 1:**
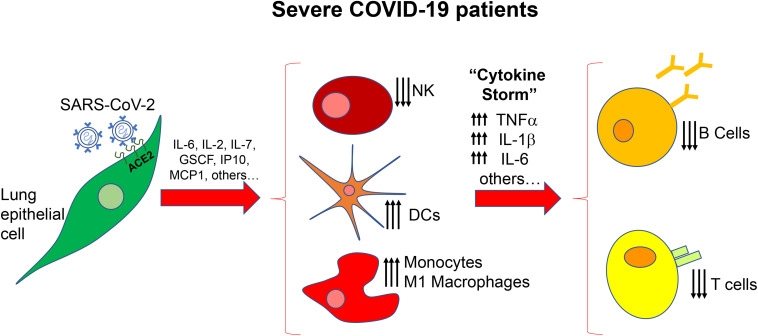
Scheme of the immune alteration in severe COVID-19 patients. After the initial secretion of inflammatory factors by lung epithelial cells there is a massive deregulation of immune cells, cytokines and chemokines resulting in increased number of dendritic cells (DCs) monocytes and macrophages and increased levels of TNFα, IL-1b, and IL-6. NK, natural killer cells.

On the other hand, it has been demonstrated that SARS-CoV-2, once inside the cells, can unleash a “cytokine storm,” through the upregulation of interleukin (IL)-2, IL-6, IL-7, granulocyte colony-stimulating factor (GSCF), interferon γ-induced protein 10 (IP10), monocyte chemoattractant protein-1 (MCP1), macrophage inflammatory protein (MIP1A) and tumor necrosis factor-alpha (TNF-α) (Figure 1) (Huang et al., 2020). Then, cytokine storm results in pulmonary oedema, prominent proteinaceous exudates, hyperplasia of pneumocytes, vascular congestion, dysfunction of air-exchange, acute respiratory distress syndrome (ARDS), acute cardiac injury and secondary infection, which may result in death (Leng et al., 2020; Metcalfe, 2020).

Nowadays, there is no specific vaccine or treatment for SARS-CoV-2, only prevention and control of the infection and supportive care, are available. Systemic corticosteroids seem to be effective in severe COVID-19 patients; but they have immunosuppressive effects (Coutinho and Chapman, 2011).

A series of approved drugs for other diseases, such as remdesivir (ebola), lopinavir–ritonavir (HIV), interferon 1β (multiple sclerosis), chloroquine and hydroxychloroquine (malaria), are under investigation. In addition, it has been suggested that cytokines involved in COVID-19 severe pathogenesis, such as IL-1 and IL-6, may be potential therapeutic targets (Mehta et al., 2020). In this scenario, MSC and its conditioned medium or extracellular vesicles (EVs) obtained from MSC strongly emerge as possible therapeutic alternatives, due to their anti-inflammatory, regenerative, pro-angiogenic, anti-fibrotic and antimicrobial capacities.

## Mesenchymal Stromal Cells

Stem cells, although in minimal quantities, are present in many tissues of the body including the lungs, and participate and control tissue renewal. Furthermore, there is a subtype of stem cells, relatively unknown until recently, called mesenchymal (stem) stromal cells (MSC), which are active sentinels and regulators in tissue homeostasis (Vizoso et al., 2019). In fact, it has been proposed that in many degenerative, autoimmune diseases (such as lupus, rheumatoid arthritis, psoriasis, etc.), or even in processes associated with aging, there could be a depletion, probably due to exhaustion, or poor function of these cells. MSC have a regulatory role in basic biological processes, such as control of cell proliferation, apoptosis, angiogenesis, oxidative stress, antimicrobial effects and, mainly, powerful anti-inflammatory properties. Therefore, the idea of restoring normal physiological function using these allogenic MSC has attracted many researchers around the world.

Mesenchymal (stem) stromal cells were firstly described as a rare population in the bone marrow (Friedenstein et al., 1970). Bone marrow MSC (BM-MSC) are the most extensively MSC studied. MSC were found in other locations, such as adipose tissue, amniotic fluid, placenta, umbilical cord, Wharton’s jelly, dermis, skeletal muscle, periosteum, lung, cartilage synovial fluid or membrane, peripheral blood, tonsil, uterus, periodontal ligament or dental pulp (Vizoso et al., 2017; Neri and Borzì, 2020). The “International Society for Cellular Therapy” established the minimum criteria required for MSC definition in 2006 as follows: (a) plastic-adherent cells; (b) simultaneously expression of stromal markers (CD29, CD44, CD73, CD90, and CD105), but negative for hematopoietic (CD45 and CD14) or endothelial (CD31 and CD34) markers, and HLA-DR surface molecules and (c) capacity to differentiate into osteoblasts, adipocytes and chondroblasts *in vitro* (Dominici et al., 2006).

Safety and efficacy of MSC in therapeutics have been demonstrated in Phases I and II, including Phase III, of clinical trials in several pathologies [for review, Samsonraj et al. (2017)]. Improved function after MSC infusions in these clinical studies has been mostly attributed to MSC capability to interact with immune cells and secreting a variety of paracrine factors, which result eventually in immunomodulation (Prockop and Oh, 2012).

## Mesenchymal Stromal Cells and Lung Diseases

It has been found a phenotype distortion and rarefication of pulmonary MSC associated to lung pathology, like in acute lung injury (ALI), chronic obstructive pulmonary disease or bronchopulmonary dysplasia, as well as effects related to aging (Foronjy and Majka, 2012; Akram et al., 2016; Gronbach et al., 2018; Reicherzer et al., 2018). However, it has also been observed that MSC can be attracted to the site of injury contributing to organ repair (Tropea et al., 2012).

Thus, MSC-based therapy is an attractive approach for treating lung diseases. In this sense, many studies based on exogenous administration of MSC have been launched with the intent of restoring physiologic cell function in the lung. These studies have shown that MSC only engraft in the injury lung sparsely and temporally. Nevertheless, MSC secretes plenty of molecules with paracrine efficacy (Zhen et al., 2008), which promote regeneration and immunoregulatory actions. MSC secreted angiopoietin 1 (ANGPT1), hepatocyte growth factor (HGF), epidermal growth factor (EGF), keratinocyte growth factor (KGF), and vascular endothelial growth factor (VEGF) have been recognized as factors promoting regeneration and protection of alveolar epithelial cells secreted by MSC (Bernard et al., 2018). In addition, MSC secrete cytokines (IL-1RA, IL-10, and TGF-β), nitric oxide and indoleamine 2,3 dioxygenase (IDO), which regulate immune cells toward an anti-inflammatory phenotype (Lee et al., 2009; Pedrazza et al., 2017). Especially relevant is the induction of MSC to a phenotype adaptation of macrophages, from the M1 inflammatory phenotype to the M2 anti-inflammatory status, which regulates inflammation, phagocytosis and enhances tissue repair.

On the other hand, MSC may display other capacities limiting lung injury. MSC can improve bacterial clearance stimulating phagocytosis activity of macrophages through the secretion of antimicrobial factors, like peptide LL-37 and lipocalin-2 (Krasnodembskaya et al., 2010; Mei et al., 2010; Gupta et al., 2012). It is also important to note the capability of MSC to prevent epithelial-mesenchymal transition of alveolar epithelial cells in the context of lung injury (Uzunhan et al., 2016).

In accordance to all of these biological observations, preclinical lung disease models of bronchopulmonary dysplasia, asthma, chronic obstructive pulmonary disease, idiopathic pulmonary fibrosis and ALI, show the therapeutic efficacy of MSC for therapeutic application (Behnke et al., 2020).

### MSC in Acute Lung Injury

Acute lung injury (ALI), caused by several insults such as viral or bacterial infections among others (Johnson and Matthay, 2010), is nowadays a global public health issue. ARDS is one frequent and evolutionary severe form of ALI, associated with a high mortality (30–40%) (Rubenfeld et al., 2005; Ranieri et al., 2012; Kreyer et al., 2016; Przybysz and Heffner, 2016). Pathogenesis of ARDS is conditioned by the dysregulation of immune response, the permeability of alveolar endothelial/epithelial barrier and the activation of coagulation (Matthay et al., 2012).

Experimental *in vivo* studies and clinical trials have been conducted to explore the therapeutic potential of MSC in ALI. Treatment based on MSC reduced alveolar permeability and lung inflammation in *in vivo* model of ALI induced by lipopolysaccharides (LPS), as well as in a human lung perfusion model (Gupta et al., 2007). In addition, MSC therapy following ALI improved tissue remodeling and lung function (Han et al., 2016). ANGPT1 and KGF were identified as the derived MSC factors responsible by these actions (McCarter et al., 2007).

Preclinical studies evaluated the treatment of ALI with MSC from BM, AT and UC (Gupta et al., 2007; Devaney et al., 2015; Hackstein et al., 2015; Li and Wu, 2015; Mao et al., 2015; Chan et al., 2016; Jackson et al., 2016; Li et al., 2016; Loy et al., 2019). In these studies, different experimental lung inflammation models were used (LPS, influenza, *Escherichia coli*, *Klebsiella*, or *Pseudomonas aeruginosa*), different application routes were tested (intravenous, intratracheal or intranasal) and different dose of cells were assayed. Beneficious biological functions were found, such as reduced lung injury, inflammation (low inflammatory cell recruitment, low pro-inflammatory cytokine production or high anti-inflammatory cytokine IL-10), lung oedema or alveolitis, reduction in bacterial load and enhance epithelial wound repair.

Interestingly, MSC are usually resistant to viral infection due to their expression of interferon (IFN) stimulated genes (ISG) such as IFITM (interferon-induced transmembrane family), IFI6, ISG15, SAT1, PMAIP1, p21/CDKN1A, and CCL2 that preempt viral infection (Wu et al., 2018). Thus, for example, it has been reported that members of the IFITM family members prevent infection before viruses can traverse the lipid bilayer of cultured cell; this has been proved for influenza A virus and SARS coronavirus (Bailey et al., 2014).

In general, clinical phase I and phase II studies demonstrate preliminary safety results in patients suffering bronchopulmonary dysplasia, asthma, chronic obstructive pulmonary disease (Ribeiro-Paes et al., 2011) idiopathic pulmonary fibrosis (Tzouvelekis et al., 2013; Chambers et al., 2014; Glassberg et al., 2017; Ntolios et al., 2018) and also in patients with ALI (Zheng et al., 2014). However, it has been reported that the intravascular administration of MSC could cause a transient increase in pulmonary pressures and lead to pulmonary edema, in susceptible patients (Chin et al., 2003).

### Clinical Trials With MSC in Patients With COVID-19

Mesenchymal (stem) stromal cells therapy effectiveness in several lung disorders was evidenced in various preclinical studies (Antunes et al., 2017; Geiger et al., 2017; Kruk et al., 2018), including ARDS (Lopes-Pacheco et al., 2020). Recently, it has been suggested the clinical treatment of H5N1 viral infections, with MSC (Chen J. et al., 2020). Thus, given the extremely serious and urgent situation of the coronavirus pandemic (SARS-CoV-2), it has been suggested that, under compassionate use protocols, MSC could be an alternative for the treatment of critically ill patients (Atluri et al., 2020).

To date, two papers have described results of COVID-19 pneumonia treatment with MSC. The first study demonstrated that the treatment with human umbilical cord (UC)-MSC was safe and showed efficacy to modulate the immune response and repaired the injured tissue of a 65-year-old female critically ill COVID-19 patient (Liang et al., 2020). MSC were administrated intravenously three times (5 × 10^7^ cells each time, every 3 days). After the second administration of UC-MSC, analytical parameters and vital signs were improved. Thereafter, the number of white blood cells and neutrophils in the patient decreased to a normal level, while the number of lymphocytes increased to their normal level.

The second study was conducted on seven patients with COVID-19 pneumonia (one displaying critically severe type, four exhibiting severe types, and the other two showing common types of the syndrome), whom received intravenous administration of 1 × 10^6^ BM-MSC cells per kilogram of weight, which were administered intravenously only one time. Three additional patients with COVID-19 severe types were enrolled as control (Leng et al., 2020). No apparent adverse effects were found after MSC injection. Patients receiving MSC showed an improvement of clinical and analytical parameters and reduced viral titers by 2–4 days after receiving MSC infusion.

Although more complete information on the patients included in the study is required (Khoury et al., 2020), interestingly, the beneficial effect of MSC was attributed to their anti-inflammatory mechanism. It is known that in COVID-19 patients, the immune system produces a cytokine storm, which includes the overproduction of immune cells and cytokines (Mehta et al., 2020). The study of Leng et al. (2020) suggests a robust anti-inflammatory effect after MSC infusion in COVID-19 patients, such as decreased of number of white blood cells and neutrophils, increased number of peripheral lymphocytes, the C-reactive protein dropped 10-fold, decreased and waning of cytokine-secreting immune cells, and increased of a group of regulatory dendritic cell (DC), decreased level of the pro-inflammatory cytokine TNF-α, and elevation of the anti-inflammatory protein interleukin-10, in peripheral blood. It is also of note that in this study infused MSC showed no expression of ACE2 and TMPRSS2, evidencing that MSC were free of SARS-CoV-2 infection. It has been also suggested that MSC therapy is more apparent in more severe COVID-19 condition (Yen et al., 2009).

However, some concerns have been reported regarding MSC therapy in COVID-19 or ARDS patients. A retrospective study of efficacy and side effects of MSC therapy in severe COVID-19 described significantly high serum level of lactate, cardiac troponin T and creatine kinase-MB after MSC therapy, suggesting a risk for patients with metabolic acidosis or coronary heart disease (Chen X. et al., 2020). Also, a higher mortality in patients treated with MSC was reported, but probably due to the more severe baseline illness in this group of treatment (Matthay et al., 2019). Regarding pulmonary function, MSC therapy showed improvement in a short-term (3–5 days) evaluation (Qu et al., 2020), but one study reported no significant change (Zheng et al., 2014).

At present, at least 30 cell-based clinical trials for treating COVID-19 are currently registered^1,2^. Of these studies, 22 are based on the use of MSC from different human origins, such as umbilical cord blood, Wharton’s Jelly or dental pulp. In addition, two clinical trials based on MSC-derived exosomes are also registered.

## MSC Secretome as New Therapeutic Strategies in Lung Diseases

Despite preliminary positive results, we have to take into account current limitations of cell therapy with MSC. For example, it is well accepted that MSC disappear within several days and their engraftment in the lung is low (Aslam et al., 2009; van Haaften et al., 2009; Liu et al., 2017), also, it is known that application of high dose of MSC resulted in vessel occlusion and pulmonary embolism (Liu et al., 2017) disseminated intravascular coagulation and respiratory and cardiovascular failure (Liao et al., 2017). It has been also reported the aggravation of lung fibrosis attributable to the excess of TGF-β1 (Schweitzer et al., 2011; Broekman et al., 2018). On the other hand, there are other several safety considerations related to MSC administration, such as cellular senescence and apoptosis, immune compatibility, tumorigenicity and the potential transmission of infections (Vizoso et al., 2017). And regarding the particular case of COVID-19 patients, it has been described that MSC exert their maximal anti-inflammatory response upon IFN-γ stimulation, however, severe COVID-19 patients show low levels of T cells, which would limit MSC activation in lungs (Shi et al., 2020).

Due to the moderate efficacy of MSC applications in the first clinical trials, research was carried out toward cellular modifications of MSC in order to improve their biological effect, such as genetic modifications, MSC preconditioning, pharmacologic modulation, or the combined application of MSC together with pharmacologic therapies (Vizoso et al., 2019). However, there is a growing trend in the scientific community to recognize that the beneficial therapeutic effect of MSC relies on the cocktail of substances that clump together under the generic term of secretome (growth factors, cytokines, or extracellular vesicles). The direct use of these substances obtained in cell cultures could be a feasible alternative to the usage of MSC.

The use of the MSC secretome offers great technical-biological strategic advantages, such as: (i) unlike cellular therapies, secretomes can be better evaluated in terms of their safety, dosage and potency, analogous to agents conventional therapeutics; (ii) secretomes can be stored without the need of the application of potentially toxic cryopreservatives; (iii) the use of products derived from the secretome is cheaper and more practical for clinical use, since the use of the secretome could avoid the time and costs associated with the expansion and maintenance of clonal cell lines; (iv) secretomes for therapies could be prepared in advance in large quantities and available for treatment when necessary and (v) MSC secretome can be tailored to specific diseases; for example, treating MSC with IFN-γ would generate a specific secretome having maximal anti-inflammatory properties that would by-pass the reduced presence of T cells in severe COVID-19 patients.

Among the components of the MSC secretome, one of the most interesting are the extracellular vesicles (EVs), which are phospholipid membrane-bound particles secreted from cells containing biomolecules such as growth factors, cytokines, lipids, DNA and various forms of RNAs. EVs, which common markers are CD9, CD63, and CD81, may be classified as exosomes (40–150 nm in diameter), microparticles (50–1,000 nm in diameter), and apoptotic bodies (500–2,000 nm in diameter). EVs represent an intercellular communication system, as well as a defense against viral attack (Rosca et al., 2017). Indeed, EVs interact with cells by mechanisms similar to those involved in viral entry. These include ligand-receptor interaction in order to trigger signal cascades, internalization of surface-bound EVs, and fusion with the cell to deliver material directly to the cytoplasmic membrane and cytosol (Pitt et al., 2016). Due to their bioactive factors MSC-EVs have proliferative, anti-apoptotic, anti-inflammatory, anti-oxidative stress, pro-angiogenic anti-fibrotic, anti-tumor, or anti-microbial activities (Vizoso et al., 2017; Mohammadipoor et al., 2018; Behnke et al., 2020). It is also important to mention that EVs contain several cytokines and growth factors including KGF, ANGPT1, EGF, HGF, and SDF-1, which enhance regeneration in lung diseases. In addition, certain proteins and nucleic acids, which have biologic protective effect, are enriched in EVs compared to their parental cells (Collino et al., 2010). Interestingly, MSC-EVs were able to limit replication and induce shedding of influenza virus, may be through the transfer of RNA to the infected cells (Khatri et al., 2018).

Extracellular vesicles have special interest for their application in therapies. They are smaller, less immunogenic and with a less membrane-bound proteins than their progenitor cells. Exosome production and storage are easier than production of parent cells. Preclinical studies demonstrated the safety of MSC-derived exosomes, with mass scalable production possible at clinically relevant doses (Vizoso et al., 2019).

Preclinical studies showed similar therapeutic efficacy of EVs or conditioned medium compared to MSC administration in various disease models like bronchopulmonary dysplasia, asthma, fibrosis and chronic obstructive pulmonary disease (Cruz et al., 2015; Srour and Thébaud, 2015; Ahn et al., 2018; Fujita et al., 2018; Mohammadipoor et al., 2018; Willis et al., 2018). Three preclinical studies in rodents have evaluated MSC-derived EVs from BM or AT, administrated via intravenous or intratracheal, as treatment for ALI (Ionescu et al., 2012; Waszak et al., 2012; Zhao et al., 2019). In these studies, anti-inflammatory effects were observed, together with antimicrobial activity, decrease of lung injury and/or increase in survival. Currently, the first clinical trial on the safety and aerosol tolerance of CMM-exosomes from adipose tissue derived (AD)-MSC has already been launched on March 2020 at Ruijin Hospital in Shanghai (China)^3^.

## Importance of the MSC Heterogeneity for Future Therapies

It has been also reported that MSC obtained from various sources differ in their biological features (Chen et al., 2015; Elahi et al., 2016) MSC heterogeneous functionality affects their proliferative capacity, cellular differentiation, angiogenesis and vasculogenesis, anti-inflammatory and anti-tumor properties (Vizoso et al., 2019).

The functional potency of MSC varies depending on the individual, and, in each individual, the capacities of these cells vary depending on the biological niche where MSC are located. Age of the donor is a recognized key factor influencing MSC capacities. It has been reported that MSC from older donors have slower proliferation rate, increased percentage of apoptotic cells, reduced immunomodulatory properties, less reparative capacity or less capability to handle oxidative stress, compared with those from younger ones (Vizoso et al., 2019). In accordance with these observations, it has been also found that in an ALI rodents model, only EVs from young donors MSC alleviated lung injury (Zhao et al., 2019). Other donor’s dependent factors influencing functional properties of MSC are obesity and general health status. There are increasing data indicating a dysfunction of MSC associated to chronic diseases such as diabetes, rheumatoid arthritis, systemic lupus erythematosus (Vizoso et al., 2019).

Heterogeneity of secretomes from MSC isolated from different tissues also was showed. Thus, for example, it has been showed that AD-MSC secreted higher amounts of pro-angiogenic molecules (matrix metalloproteinases (MMPs) (Amable et al., 2014) or VEGF (Hsiao et al., 2012), compared with other MSC, such as BM-MSC; whereas UC-MSC secreted high levels of immunomodulatory factors, such as IL-6, -7 and -10, PDGF-AA and TGF-α (Amable et al., 2014).

The concept of heterogeneity of MSC seems to extend to their therapeutic interest in acute lung diseases. Thus, a recent systematic review study concluded that BM- and UC-MSC are more effective in decreasing mortality in pre-clinical models of acute lung injury (McIntyre et al., 2016). In addition, the heterogeneity of MSC has also been described in terms of their susceptibility to viral attack. As discussed above, MSC are generally resistant to viral infection. However, it has been described that that human BM-MSC are permissive to avian influenza A (H5N1) infection, losing viability and immunoregulatory activities (Li et al., 2016). This occurs because BM-MSC express, on cell surface, influenza virus receptors and can support replication of both avian H1N1and H9N5 influenza strains (Khatri et al., 2010; Khatri and Saif, 2013).

Thus, MSC heterogeneity mirrors the diversity of environments present in the natural stem cell niches, which are consequence of the broad cellular communities that have variable chemical and mechanical conditions. This lead us to consider the existence of MSC with special capacities according to their biological environment. In this context, it is reasonable to consider the existence of MSC accustomed to regulating homeostasis in tissues exposed to external aggressions. Although AD-, BM, and UC-MSC have been the most widely used MSC in regenerative medicine, two sources appear to be candidates for hosting those special MSC: the oral cavity and the uterine cervix.

The oral cavity harbor a huge universe of more than 700 species of microorganism (Aas et al., 2005). Oral MSC have been isolated from different anatomical structures at this location [periodontal ligament, dental pulp, exfoliated deciduous teeth (SHED), gingival, apical papilla, dental follicle, bone marrow from the alveolar bone proper and periapical cyst]. Apart from their remarkable regenerative potential, it has been indicated that oral MSC possess the capacity to interact with an inflammatory microenvironment (Zhou L.-L. et al., 2020). Gingival MSC (GMSC) have a high inflammatory resistance. Experimental studies demonstrated that inflammatory stimuli of GMSC induced a weak inflammatory response and did not affect to their regenerative capacity (Zhou et al., 2017). It has also been demonstrated that the stemness and differentiation potential of GMSC is maintained under a proinflammatory cytokines stimulation (Zhang et al., 2017). Interestingly, it has also been found that GMSC or dental pulp MSC (DPSC), showed variations in the toll-like receptors (TLR) expression profile depending if they are under inflamed conditions (Fawzy-El-Sayed et al., 2016; Fawzy El-Sayed et al., 2016, 2017). This is relevant because TLR expression affect MSC proliferation, migration, differentiation potential, interaction with inflammatory environment. In addition, these variations in the TLR expression profile affect the recognition ability of MSC for different pathogens, as well as damage-associated molecular patterns under inflammation (Baik et al., 2008).

On the other hand, the immunoregulatory effects of oral MSC have been demonstrated in *in vivo* models of inflammatory, autoimmune or allergic diseases. On the basis of all of these experimental evidences oral MSC have recently considered as “immunomodulatory masters” (Zhou L.-L. et al., 2020).

The vagina hosts an acid and pro-inflammatory milieu, in which bacteria, yeasts and other microorganisms are present. This environment is exposed to the disruption of its homeostasis by the penetration of potentially dangerous elements, like some strains of the papillomavirus family, most usually during sexual intercourse. A special type of MSC has been found in the transformation zone from human uterine cervix might to display protective effect in these circumstances (Schneider et al., 2016).

Human uterine cervical stem cells (hUCESC) have a high proliferative rate (Eiró et al., 2014) and its secretome has potent regenerative (Bermudez et al., 2015; Sendon-Lago et al., 2019), anti-inflammatory (Eiró et al., 2014; Bermudez et al., 2016), anti-tumor (Eiró et al., 2014) and anti-microbial capacities (Bermudez et al., 2015; Schneider et al., 2018). The comparison of cytokines profile from secretome of AD-MSC (one of the most MSC type used in clinical trials) and of hUCESC shows that the latter has higher levels of cytokines with recognized effect in regenerative (TIMP-1, TIMP-2, FGF-6, FGF-7, uPAR, and HGF) (Bermudez et al., 2015; Sendon-Lago et al., 2019), anti-tumoral (FLT-3 ligand, LAP, LIGHT, and IP-10) (Eiró et al., 2014), and anti-inflammatory (IL-13 and NT-3) (Eiró et al., 2014; Bermudez et al., 2016) processes.

The immunoregulatory capacity of its secretome may be of interest against the inflammatory mechanism associated with the respiratory distress syndrome in patients affected of SARS-CoV-2. Thus, for example, it was reported that conditioned medium of hUCESC reduce leukocyte infiltration in ocular tissues after endotoxin-induced uveitis, similar to that achieved with dexamethasone treatment (Bermudez et al., 2016). It also inhibits and reverse monocyte differentiation to macrophages, greater than inhibition and reversion achieved with AD-MSC (Eiró et al., 2014), and also reduce tissue levels of IL-6, IL-8, IFN-γ, TNF-α, MCP-1, or MIP-1α mRNA, as well as increase of the anti-inflammatory interleukin IL-10 (at levels comparable to those achieved with dexamethasone treatment) (Bermudez et al., 2015, 2016; Sendon-Lago et al., 2019).

It is important to note the effect of hUCESC reducing IL-6, as IL-6 has been proposed to be the main driver of the COVID-19 cytokine storm. In fact, high levels of IL-6 correlates with respiratory failure and it has become an important therapeutic target for COVID-19 (Moore and June, 2020). For all these reasons, hUCESC could be a new option for the treatment of COVID-19.

## Conclusion and Future Perspectives

The coronavirus pandemic (SARS-CoV-2) demands new therapeutic alternatives. MSC can be an alternative if we consider their anti-inflammatory, regenerative, angiogenic, and even their antimicrobial capacity. Furthermore, clinical trials using this cell therapy have generally demonstrated safety and efficacy. Preliminary data point to a therapeutic effect of MSC in patients with COVID-19. The effect seems based on the MSC’s ability to curb the cytokine storm caused by COVID-19. Other clinical studies are currently underway, and it has been also suggested that the administration of MSC under compassionate use protocols may be an alternative to the treatment of critically ill patients (Atluri et al., 2020).

Nevertheless, considering the limits or deficiencies in the generation of new drugs, we must be fully aware of the importance of continuing to investigate about this great paradigm of science and medicine, which is the world of stem cells and regenerative medicine. From these approaches, based on the very balance that nature offers, new ideas and initiatives have to come out that help us face this and other future health crises. This crisis forces us, more than ever, to rush and integrate the possibilities of science and technologies available for the solutions of today and tomorrow.

Mesenchymal (stem) stromal cells secretome-derived products can reproduce the therapeutic effects of MSC in lung injury. The use of conditioned medium or EVs may avoid security inconveniences associated with the administration of MSC and may be administered on different formulations. Thus, it has been suggested that MSC-secretome can be formulated as both inhalable and injectable dosage forms (Bari et al., 2019), remaining stable in the blood until distribution to the lungs (Morishita et al., 2017). In addition, considering in treating a pandemic, the costs of MSC-secretome seem probably lower compared to monoclonal antibody therapy.

However, different limitations must be resolved. The choice of the most optimal MSC, taking into account both heterogeneity among donors and among the different biological niches. The massive obtention of the products derived from its secretome will also be necessary, possibly through the immortalization or other genetic manipulations of the most appropriate MSC, the use of bioreactors that allow its growth in 3-D, together with the most optimal culture conditions (as pH, O_2_ tension, type of media and supplements, substrates and extracellular cues, inflammatory stimuli, etc.) and the use of adequate functional tests of these obtained biological products before clinical application. In addition, the incorporation of artificial intelligence tools can contribute to the proper integration of all this new emerging information.

## Author Contributions

NE, JC, MF, and LC prepared the figures and manuscript. NE and FV designed the project and wrote the manuscript. All authors reviewed the manuscript.

## Conflict of Interest

FV and NE are co-inventors of a patent (“Human uterine cervical stem cell population and uses thereof”) owned by GiStem Research, of which NE, LC, and FV are shareholders. The funding sponsors had no role in the design of this review, in the collection, analyses, or interpretation of data, in the writing of the manuscript, or in the decision to publish the results. The remaining authors declare that the research was conducted in the absence of any commercial or financial relationships that could be construed as a potential conflict of interest.

## Footnotes

1 http://www.chictr.org.cn

2 https://clinicaltrials.gov

3 https://clinicaltrials.gov/ct2/show/NCT04313647
